# Progressive Retinal and Neurologic Findings in a Family With Neuropathy, Ataxia, and Retinitis Pigmentosa Syndrome

**DOI:** 10.1177/24741264261463171

**Published:** 2026-07-08

**Authors:** Faizan Naveed, Salem Abu Al-Burak, Erfan Hashemi, Brian G. Ballios, Shaheer Aboobaker

**Affiliations:** 1 Faculty of Medicine, University of Ottawa, Ottawa, ON, Canada; 2 Schulich School of Medicine & Dentistry, Western University, London, ON, Canada; 3 Toronto Retina Institute, North York, ON, Canada; 4 Department of Ophthalmology & Vision Science, University of Toronto, Toronto, ON, Canada

**Keywords:** neuropathy, ataxia, and retinitis pigmentosa, MT-ATP6, m.8993T>G, heteroplasmy, retinitis pigmentosa, case series

## Abstract

**Purpose:**

To describe the longitudinal ophthalmic findings of a maternally related triad with neuropathy, ataxia, and retinitis pigmentosa and explore potential relationships between phenotype and heteroplasmy levels.

**Methods:**

A mother, daughter, and son with genetically confirmed neuropathy, ataxia, and retinitis pigmentosa due to the m.8993T>G MT-ATP6 variant (heteroplasmy levels: 38.8%, 76.8%, and 87%, respectively) were followed for 5 years. Assessments included best-corrected visual acuity, slitlamp examination, dilated fundus examination, optical coherence tomography, widefield color fundus imaging, fundus autofluorescence, full-field and multifocal electroretinography, and Goldmann visual field testing.

**Results:**

Longitudinal evaluation demonstrated marked intrafamilial variability. Patient 1 demonstrated widespread ellipsoid zone loss and rod–cone dysfunction; Patient 2 exhibited retinal thinning and cone-predominant dysfunction. Patient 3 showed initially normal retinal structure with subtle fundus autofluorescence changes and mild asymmetric functional loss.

**Conclusions:**

Despite harboring the same pathogenic MT-ATP6 variant, affected family members exhibited substantial variability in ophthalmic disease severity. Clinical manifestations did not correlate with blood heteroplasmy levels, highlighting the importance of longitudinal multimodal ophthalmic surveillance in patients with neuropathy, ataxia, and retinitis pigmentosa.

## Introduction

Neuropathy, ataxia, and retinitis pigmentosa is a rare inherited mitochondrial neurodegenerative disorder initially described by Holt et al in 1990.^1,2^ It is linked to a thymine-to-guanine point mutation at nucleotide 8993 in the mitochondrial MT-ATP6 gene (m.8993T>G). Less common pathogenic variants include a thymine-to-cytosine substitution at the same site (m.8993T>C) or a guanine-to-adenine substitution at nucleotide 14459 in the MT-ND6 gene (m.14459G>A). These point mutations impair the structure and function of ATP synthase, resulting in reduced ATP production and progressive multisystem dysfunction, often presenting in early childhood.^3,4^ The age at onset and severity of clinical manifestations vary widely among affected individuals and may correlate with the degree of heteroplasmy.^5^ However, the relationship between heteroplasmy and disease severity is not perfect. Furthermore, heteroplasmy measurements are ideally obtained from affected tissues and interpreted in conjunction with longitudinal surveillance of the patients and their manifestations.^6,7^

Clinically, neuropathy, ataxia, and retinitis pigmentosa encompasses a wide range of ophthalmologic and neurologic manifestations. Ophthalmic involvement is common and may serve as a precursor to neurologic progression.^8,9^ Early ophthalmic manifestations include nyctalopia, progressive constriction of peripheral vision, and retinal dystrophy resembling retinitis pigmentosa.^10^ Neurologic features commonly include cerebellar ataxia, peripheral neuropathy, proximal muscle weakness, seizures, and cognitive impairment.^9,11^

The m.8993T>G variant in MT-ATP6 is associated with a high risk of severe disability when blood heteroplasmy levels exceed approximately 60% to 70%; however, clinical severity often does not correlate directly with blood heteroplasmy measurements.^7,12^ Accordingly, blood heteroplasmy levels should be interpreted in conjunction with comprehensive clinical, functional, and imaging data, rather than in isolation.^7,13^ This report of a 3-member maternal lineage highlights the variable expression and multisystem nature of neuropathy, ataxia, and retinitis pigmentosa, even among individuals harboring the same pathogenic variant.

## Case Descriptions

### Case 1

A 57-year-old woman with clinically and genetically confirmed neuropathy, ataxia, and retinitis pigmentosa syndrome due to the m.8993T>G MT-ATP6 variant (blood heteroplasmy level 38.8%) was referred in 2020 for evaluation of progressive visual decline. She had initially been diagnosed in 1990 based on a strong maternal family history and retinal changes, with subsequent genetic confirmation of the diagnosis. The patient was the first identified carrier in her family and was the mother of 2 surviving children. Two additional children died in early childhood, and 1 pregnancy resulted in a stillbirth, findings consistent with maternal inheritance of a mitochondrial disorder. No consanguinity was reported.

The patient first noted difficulties in night vision and peripheral visual field loss around 2005, with gradual progression to central visual involvement. Her systemic history was notable for hypothyroidism, peripheral neuropathic pain, and mild gait imbalance. At presentation, her medications included levothyroxine, pregabalin, carnitine, lipoic acid, and creatine.

**Figure 1.**
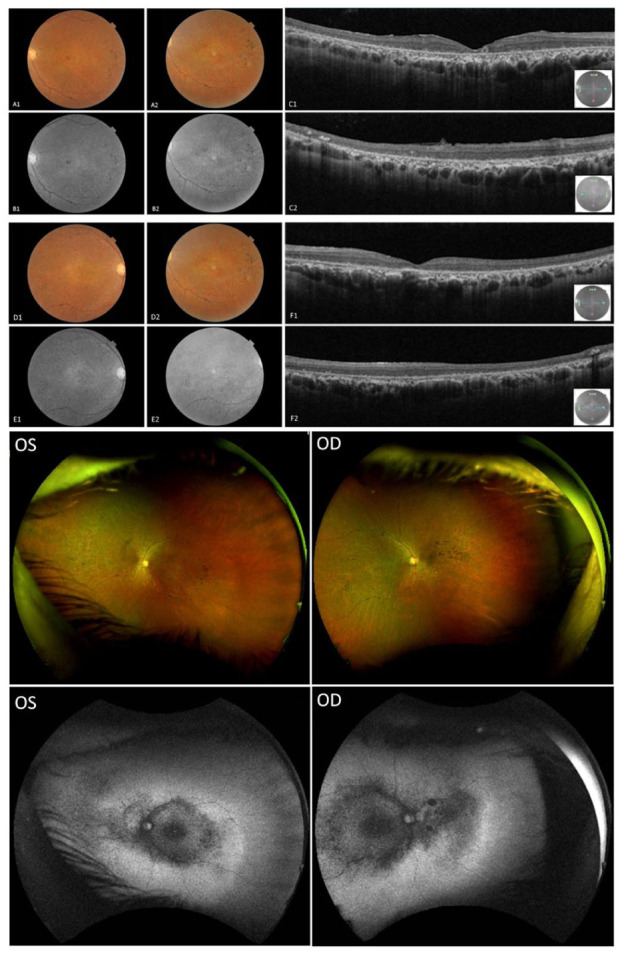
Multimodal retinal imaging of Patient 1. Optical coherence tomography, widefield color fundus photography, and fundus autofluorescence (FAF) demonstrating outer retinal and retinal pigment epithelium atrophy, ellipsoid zone loss, midperipheral pigmentary changes, and diffuse autofluorescence abnormalities. Image pairs (A1–A2, B1–B2, C1–C2, D1–D2, E1–E2, and F1–F2) show interval changes between September 10, 2020 (age 57 years) and April 22, 2024 (age 61 years). Widefield Optos color fundus and FAF images of both eyes were obtained in April 2024.

At referral, best-corrected visual acuity (BCVA) was 20/50 OD and 20/40 OS. Slitlamp examination was unremarkable, with no evidence of posterior subcapsular cataract or epiretinal membrane (ERM). Optical coherence tomography (OCT) revealed diffuse outer retinal and retinal pigment epithelium (RPE) atrophy with central ellipsoid zone (EZ) loss in the right eye and parafoveal EZ discontinuity with small preserved islands and mild outer nuclear layer thinning in the left eye. No cystoid macular changes were observed. Color fundus imaging showed midperipheral pigmentary alterations without classic bone-spicule pigmentation. Widefield imaging demonstrated diffuse vascular attenuation, optic disc pallor, and confluent central hypoautofluorescence with surrounding granular hyperautofluorescence and peripheral patchy hypoautofluorescence, findings consistent with widespread RPE and photoreceptor loss (Figure 1).

During 2 years of follow-up, BCVA declined slightly before stabilizing at 20/40 OU. OCT and fundus autofluorescence (FAF) findings remained stable. The patient denied hearing loss or vertigo but reported chronic musculoskeletal pain and intermittent gait imbalance. Management involved annual ophthalmic monitoring, low-vision rehabilitation, and mitochondrial cofactor supplementation.

**Figure 2.**
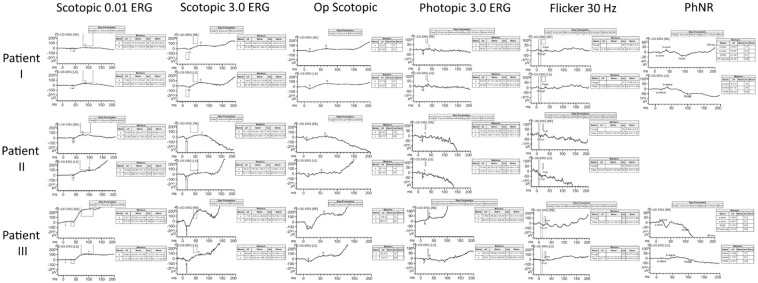
Full-field electroretinography findings in Patients 1–3. Scotopic, photopic, 30 Hz flicker, and photopic negative response recordings showing generalized rod–cone dysfunction in Patient 1, preserved waveform morphology with reduced amplitudes in Patient 2, and marked interocular asymmetric dysfunction in Patient 3, characterized by reduced amplitudes and prolonged implicit times in scotopic and photopic responses, respectively.

**Figure 3.**
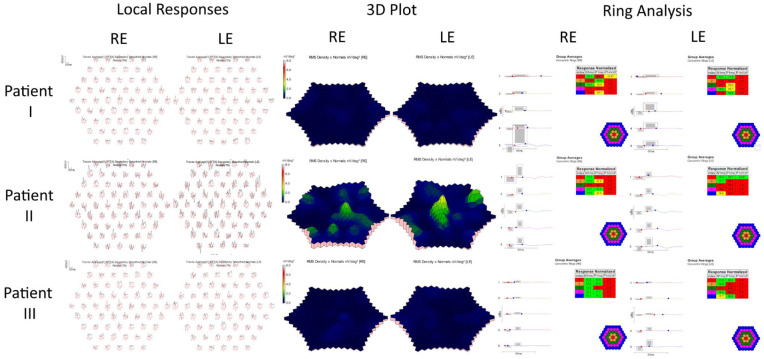
Multifocal electroretinography (mfERG) findings in Patients 1–3. Representative local responses, 3D response density plots, and ring analyses are shown. mfERG responses were absent in all patients, with Patient 2 demonstrating a questionable residual foveal response in the left eye, consistent with significant cone–rod dystrophy and advanced macular involvement.

**Figure 4.**
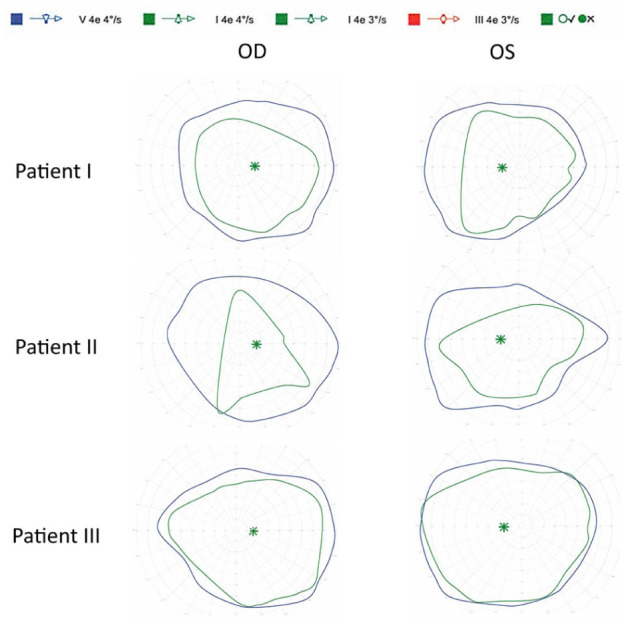
Goldmann visual field testing findings in Patients 1–3. Goldmann visual fields obtained using the I4e and V4e isopters demonstrate relative preservation of peripheral visual field function across all 3 patients. Patient 1 exhibited full peripheral isopters. Patient 2 demonstrated preserved peripheral fields. Patient 3 showed preserved peripheral field boundaries with a broader I4e isopter in the right eye and mild constriction in the left eye, corresponding to the interocular asymmetry observed on electroretinography.

Full-field electroretinography (ERG) demonstrated symmetric generalized rod–cone dysfunction with reduced amplitudes and delayed implicit times. Multifocal ERG (mfERG) responses were absent across all macular regions bilaterally. Goldmann visual field testing revealed full peripheral isopters, indicating preserved peripheral function (Figures 2–4).

### Case 2

A 28-year-old woman, the daughter of Patient 1, with genetically confirmed neuropathy, ataxia, and retinitis pigmentosa syndrome was evaluated for episodic decreases in visual acuity and progressively worsening peripheral and night vision. Unlike her mother, she was diagnosed during infancy. Genetic testing confirmed the m.8993T>G MT-ATP6 mutation, with a blood heteroplasmy level of 76.8% compared with a previously reported heteroplasmy level of 38.5% in lymphoblasts.

**Figure 5.**
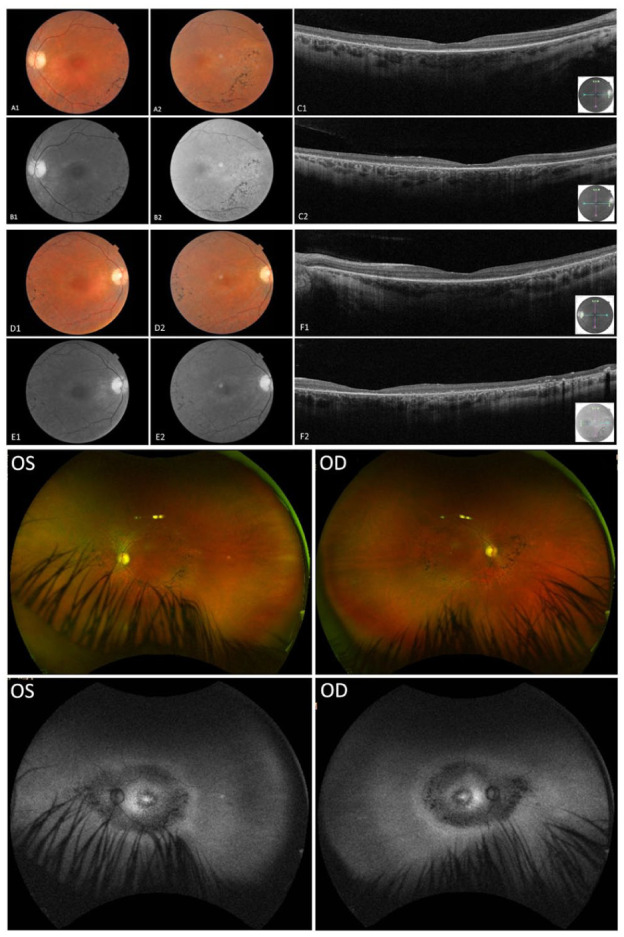
Multimodal retinal imaging of Patient 2. Optical coherence tomography, widefield color fundus photography, and fundus autofluorescence (FAF) images demonstrating parafoveal ellipsoid zone disruption, retinal thinning, bone-spicule pigmentation, vascular attenuation, and widespread retinal pigment epithelium abnormalities associated with central and peripheral autofluorescence abnormalities. Image pairs (A1–A2, B1–B2, C1–C2, D1–D2, E1–E2, and F1–F2) show interval changes between September 10, 2020 (age 28 years) and April 22, 2024 (age 32 years). Widefield Optos color fundus and FAF images of both eyes were obtained in April 2024.

At the initial assessment in August 2020, the patient was taking lipoic acid, carnitine, creatine, vitamin B2, vitamin D, and coenzyme Q10. BCVA was 20/50 OD and 20/40 OS. Slitlamp examination revealed no posterior subcapsular cataract or ERM. OCT demonstrated generalized retinal thinning with parafoveal EZ disruption and preserved foveal contour. Fundus photographs revealed midperipheral bone-spicule pigmentation, waxy optic disc pallor, and diffuse RPE alteration. Widefield color imaging showed vascular attenuation and extensive pigmentary changes extending into the midperipheral and far-peripheral retina. FAF revealed central hypoautofluorescence surrounded by granular autofluorescence abnormalities and patchy peripheral hypoautofluorescence, findings consistent with diffuse RPE dysfunction (Figure 5).

Clinical observation and low-vision rehabilitation were recommended. Neurologic evaluation identified epilepsy characterized by focal seizures (4–5 episodes per week, often related to stress), chronic migraines, and episodic dizziness. Neurologic examination demonstrated mild cerebellar dysfunction, including truncal instability and dysdiadochokinesia, as well as subtle distal sensory deficits. Neuropsychological testing revealed mild intellectual disability with executive dysfunction. Audiometry testing performed in 2021 was normal.

At 1-year follow-up (age 29 years), BCVA remained stable. However, by 2022 (age 30 years), the patient reported worsening central vision and photophobia, with BCVA declining to 20/70 OD and 20/50 OS. OCT demonstrated progressive retinal thinning. By April 2024 (age 32 years), BCVA had further decreased to 20/80 OD and 20/70 OS, although OCT and FAF findings appeared stable compared with those of the previous examination. The patient demonstrated multisystem involvement, with epilepsy, migraines, ataxia, cognitive impairment, and mild neuropathic symptoms, but remained ambulatory.

Full-field ERG revealed symmetric responses with preserved scotopic waveform morphology but reduced amplitudes. Cone-isolated photopic LA 3.0 responses were not measurable above background noise, and 30 Hz flicker responses were markedly reduced in amplitude and delayed in implicit time. mfERG responses were absent bilaterally, with only a questionable foveal response in the left eye, likely attributable to noise. Overall, the electrophysiologic findings were consistent with bilateral cone–rod dystrophy with greater cone involvement. Goldmann visual field testing showed relative preservation of peripheral visual field function (Figures 2–4).

### Case 3

A 17-year-old boy, the half-brother of Patient 2 and son of Patient 1, was evaluated for ophthalmic surveillance owing to genetically confirmed neuropathy, ataxia, and retinitis pigmentosa syndrome. Genetic testing confirmed the m.8993T>G MT-ATP6 mutation, with a blood heteroplasmy level of 87%.

**Figure 6.**
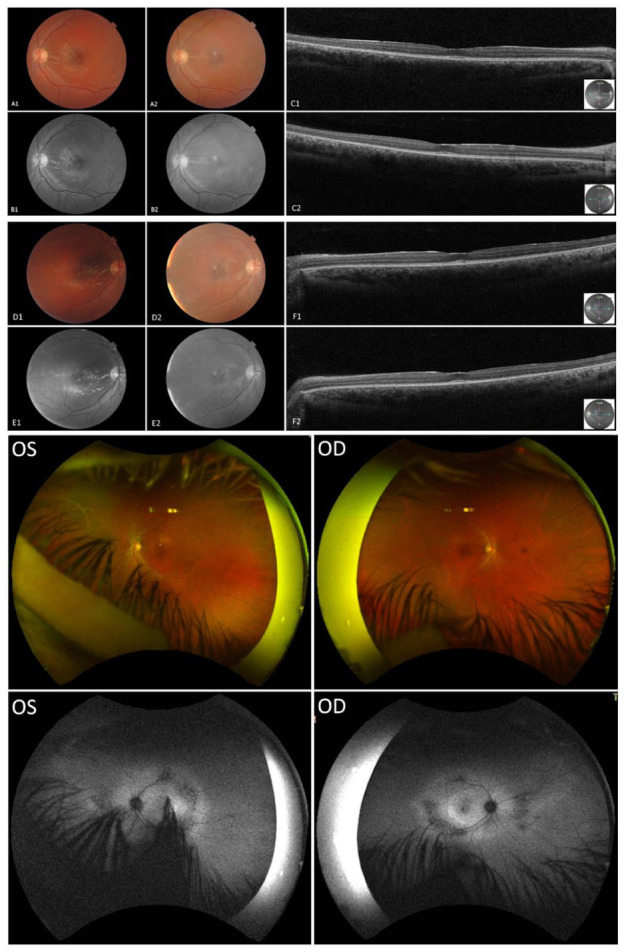
Multimodal retinal imaging of Patient 3. Optical coherence tomography, fundus autofluorescence (FAF), and widefield images showing preserved retinal architecture with only subtle central autofluorescence reduction. Image pairs (A1–A2, B1–B2, C1–C2, D1–D2, E1–E2, and F1–F2) show interval changes between August 27, 2020 (age 17 years) and April 22, 2024 (age 21 years). Widefield Optos fundus and FAF images of both eyes were obtained in April 2024.

At baseline in 2020, the patient was taking montelukast, cetirizine, fluticasone propionate, salbutamol, vitamins B–E, and coenzyme Q10. He was visually asymptomatic apart from mild light sensitivity and occasional floaters and denied nyctalopia. BCVA was 20/20 OU. Slitlamp examination revealed no posterior subcapsular cataract or ERM, and both slitlamp and fundus examinations were otherwise unremarkable. OCT and FAF demonstrated normal retinal architecture and no RPE changes. Widefield imaging showed preserved retinal structure with largely uniform autofluorescence and only subtle central signal reduction, suggestive of minor disease progression (Figure 6).

At the 2021 follow-up visit (age 18 years), visual function remained stable, with a BCVA of 20/20 OU and no changes on OCT or FAF. The patient also reported a decrease in the frequency of floaters. By 2023 (age 20 years), FAF revealed early subtle midperipheral hypoautofluorescence, although OCT findings and BCVA remained stable. The patient continued to deny nyctalopia or peripheral visual field loss. Neurologic evaluation at that time revealed mild ataxia, intermittent gait imbalance, exertional fatigue, dysdiadochokinesia, and slowing of fine motor movements.

At the follow-up in 2024 (age 21 years), BCVA remained 20/20 OU, with stable findings on OCT and fundus examination. FAF demonstrated mild progression of midperipheral hypoautofluorescence without new macular changes.

ERG testing was reliable and revealed interocular asymmetry. The right eye demonstrated near-normal scotopic responses with delayed B-wave peaks and mildly reduced photopic amplitudes. The 30 Hz flicker response was present but reduced in amplitude and delayed in implicit time. The left eye showed preserved scotopic waveform morphology but reduced amplitudes and delayed implicit times; photopic responses were not measurable above background noise. mfERG responses were absent across all tested macular regions bilaterally. Goldmann visual field testing showed preserved peripheral visual field boundaries, with a broader I4e isopter in the right eye and mild constriction in the left eye, mirroring the electrophysiologic asymmetry observed on ERG (Figures 2–4).

## Conclusions

This case series provides a rare longitudinal characterization of 3 maternally related individuals with neuropathy, ataxia, and retinitis pigmentosa syndrome, highlighting the broad phenotypic spectrum and variable disease progression associated with the m.8993T>G mutation in the MT-ATP6 gene. In a prior case series of a family triad with the same mutation (m.8993T>G), consisting of a 57-year-old mother and her 2 adult children (a 38-year-old woman and a 30-year-old man), heteroplasmy levels of 74% and 86% were reported in peripheral blood for the daughter and the son, respectively. Ophthalmic findings included bull’s eye maculopathy on FAF, annular hyperautofluorescent rings, and rod–cone dysfunction on full-field ERG across all affected family members, with considerable variability in clinical severity across individuals.^14^

In contrast, the patients in our series demonstrated parafoveal EZ loss, progressive visual field abnormalities, and notably slower progression of structural changes, with greater preservation of BCVA throughout follow-up. Progression of ophthalmic findings in our series often paralleled worsening neurologic involvement, although subtle retinal changes were also identified in the absence of significant neurologic decline. These findings reinforce the marked intrafamilial variability associated with neuropathy, ataxia, and retinitis pigmentosa syndrome and suggest that phenotypic heterogeneity may be the rule rather than the exception.^15^

All patients in this series were genetically confirmed to harbor the pathogenic m.8993T>G MT-ATP6 variant, with varying blood heteroplasmy levels. Prior studies have established a pathogenic threshold for this mutation at approximately 60% to 70% heteroplasmy in blood, above which patients are more likely to manifest systemic features such as ataxia, epilepsy, and visual loss.^7^ However, the relationship between heteroplasmy and clinical severity remains imperfect. The marked variability observed between the siblings and their mother highlights the influence of heteroplasmy, tissue-specific distribution of mutated mitochondrial DNA, and possibly other genetic or environmental modifiers in shaping clinical outcomes.^16^

Ophthalmic involvement was an early and consistent feature across all 3 patients in this case series. Patient 1 experienced progressive visual decline over more than 2 decades, with classic features of mitochondrial retinopathy.^9^ Patient 2 followed a similar trajectory but exhibited more rapid deterioration in visual acuity by her early 30s, reflecting earlier systemic involvement and a higher blood heteroplasmy level. In contrast, Patient 3, despite a blood heteroplasmy level of 87%, maintained normal central visual acuity and preserved retinal architecture on OCT, with only subtle emerging signs of midperipheral retinal degeneration.

This differential expression is consistent with prior observations that retinal manifestations in neuropathy, ataxia, and retinitis pigmentosa may not correlate with neurologic involvement or progress in parallel with systemic disease.^2,9^ These findings underscore the importance of early diagnosis and longitudinal multimodal ophthalmic surveillance, regardless of heteroplasmy level or neurologic disease severity.

Functional testing revealed shared and distinct electrophysiologic features across the 3 patients. Patient 1 demonstrated symmetric generalized rod–cone dysfunction with preserved waveform morphology but markedly reduced amplitudes and delayed implicit times, consistent with a rod–cone or cone–rod dystrophy pattern. Patient 2 also showed symmetric bilateral dysfunction with relatively greater cone involvement, including markedly reduced or absent photopic and 30 Hz flicker responses accompanied by reduced scotopic amplitudes, findings consistent with cone–rod dystrophy. Patient 3 exhibited clear interocular asymmetry, with near-normal scotopic responses and mildly reduced photopic amplitudes in the right eye, but markedly reduced amplitudes and delayed responses in the left eye, again consistent with cone–rod dystrophy.

Goldmann visual field findings mirrored the ERG patterns. Patients 1 and 2 showed preserved peripheral isopters despite substantial ERG dysfunction, whereas Patient 3 exhibited preserved peripheral boundaries with a broader I4e isopter in the right eye and mild constriction in the left eye, mirroring the observed ERG asymmetry. These differences likely reflect varying disease stage, macular involvement, and heteroplasmy distribution. However, localized variability in mitochondrial dysfunction may also contribute to phenotypic variability, and the underlying mechanisms remain incompletely understood.

Neurologically, this series illustrates the broad spectrum of involvement associated with neuropathy, ataxia, and retinitis pigmentosa. Patient 2 displayed classic features, including epilepsy, cerebellar dysfunction, cognitive impairment, and mild neuropathy, aligning with previous reports indicating that individuals with blood heteroplasmy levels exceeding 70% commonly develop seizures and ataxia.^9^ Patient 3 showed more subtle abnormalities, such as dysdiadochokinesia, imbalance, and exertional fatigue, despite an even higher blood heteroplasmy level. Patient 1 remained largely neurologically stable until her sixth decade, later developing mild imbalance and sensory symptoms after a hemorrhagic stroke. This delayed systemic involvement is consistent with the age-dependent penetrance and phenotypic evolution characteristic of mitochondrial disorders (Table 1).^9,16^

**Table 1.** Comparative Summary for Patients I–III.

Patient	Genetics	OCT/FAF	ERG	VF	Systemic
Patient I (Mother)	m.8993T>G MT-ATP6; 38.8% heteroplasmy (blood)	Diffuse EZ loss OD, parafoveal EZ disruption with foveal islands OS; stable over time	Generalized rod and cone dysfunction with reduced amplitudes, delayed implicit times, and absent mfERG	Peripheral field preserved; pericentral defect not captured by kinetic protocol	Chronic pain, intermittent imbalance, mild sensory complaints; no major neurologic deficits
Patient II (Daughter)	m.8993T>G MT-ATP6; 76.8% heteroplasmy (blood), 38.5% in cultured lymphoblasts	Generalized retinal thinning with parafoveal EZ disruption; bone spicules, RPE atrophy; progressive thinning	Symmetric cone–rod dysfunction with reduced scotopic amplitudes, absent photopic and flicker responses, and absent mfERG	Peripheral preservation with mild I4e contraction; prior automated testing showed central sensitivity loss	Epilepsy, migraines, mild ataxia, cognitive impairment; multisystem involvement
Patient III (Son)	m.8993T>G MT-ATP6; 87% heteroplasmy (blood)	Normal OCT initially; subtle mid-peripheral FAF hypoautofluorescence by 2023; preserved fovea	Asymmetric dysfunction: OD near-normal scotopic with delayed B-wave; OS reduced scotopic and absent photopic; mfERG absent bilaterally	Peripheral boundaries preserved; OD broader I4e, OS mildly constricted, mirroring ERG asymmetry	Mild ataxia, exertional fatigue, fine motor slowing; otherwise preserved function; early systemic involvement

Abbreviations: BCVA, best-corrected visual acuity; CF, count fingers; ERG, electroretinography; EZ, ellipsoid zone; FAF, fundus autofluorescence; HM, hand motion; I4e, Goldmann visual field stimulus I4e; mfERG, multifocal electroretinography; MT-ATP6, mitochondrial ATP synthase membrane subunit 6; m.8993T>G, MT-ATP6 point mutation at nucleotide 8993; RPE, retinal pigment epithelium; VF, visual fields.

Importantly, this case series highlights the value of a multidisciplinary, longitudinal care model for patients with mitochondrial disease. All 3 patients received coordinated follow-up in ophthalmology, neurology, genetics, and psychiatry, with interventions including low-vision support, anti-seizure therapy, and metabolic counseling. Such comprehensive care may help mitigate disease burden, enhance functional capacity, and facilitate early identification and prevention of complications.

Moreover, our findings support the emerging notion that fluctuations in visual acuity among patients with mitochondrial retinopathies may not necessarily reflect structural retinal improvement but may be attributable to functional adaptation and refractive variability.^17,18^

Several limitations of this case series should be noted. First, heteroplasmy was assessed only in peripheral blood (and in lymphocytes in 1 case), which may not accurately reflect mutation load in the retina or brain. Therefore, correlations between heteroplasmy level and clinical phenotype should be interpreted with caution. Second, the functional testing modalities used also have inherent limitations. Although Goldmann kinetic visual field testing using the I4e and V4e targets reliably characterizes peripheral boundaries, it may fail to detect pericentral defects. In addition, mfERG responses were largely absent, limiting the ability to quantify residual macular cone function.

In conclusion, this case series demonstrates that elevated blood heteroplasmy levels do not uniformly predict early disease onset or severe disease in neuropathy, ataxia, and retinitis pigmentosa syndrome. By providing a multigenerational perspective that integrates multimodal retinal imaging, ERG, and visual field assessment, this study highlights the substantial phenotypic variability associated with the m.8993T>G MT-ATP6 variant. These findings underscore the importance of ongoing ophthalmic surveillance for monitoring disease progression in patients with neuropathy, ataxia, and retinitis pigmentosa syndrome.
